# Internet-based cognitive behavioral intervention for adolescents with anxiety disorders: a study protocol for a parallel three armed randomized controlled trial

**DOI:** 10.1186/s13063-024-08511-0

**Published:** 2024-10-13

**Authors:** Helene Skaarnes, Nikita Marie Sørensen, Anders House Wisnewski, Johanne Jeppesen Lomholt, Mikael Thastum, Lauren McLellan, Kim Mathiasen

**Affiliations:** 1 Research Unit for Digital Psychiatry, Center for Digital Psychiatry, Mental Health Services in the Region of Southern Denmark, Heden 11, Odense, 5000 Denmark; 2 Department of Clinical Research, Faculty of Health Sciences, University of Southern Denmark, Odense C, Denmark; 3 Department of Psychology and Behavioral Sciences, School of Business and Social Sciences, Aarhus University, Bartholins Allé 11, Aarhus, 8000 Denmark; 4 Center for Emotional Health, Department of Psychology, Macquarie University, Wallumattagal Campus, North Ryde, New South Wales 2109 Australia

**Keywords:** Adolescents, Anxiety disorders, Cognitive behavioral therapy, Internet-based, Digital health, Study protocol

## Abstract

**Background:**

Anxiety disorders are among the most prevalent mental health concerns affecting children and adolescents. Despite their high prevalence, statistics indicate that fewer than 25% of individuals in this demographic seek professional assistance for their condition. Consequently, there is a pressing need to develop innovative interventions aimed at improving treatment accessibility.

**Objectives:**

This study aims to assess the effectiveness of Internet-delivered Cognitive Behavioral Therapy (iCBT) for adolescents with anxiety, with a specific emphasis on involving parents in the treatment process.

**Methods:**

The study is structured as a parallel three-armed randomized controlled trial, comparing Internet-delivered Cognitive Behavioral Therapy (iCBT) with planned feedback, iCBT with on-demand feedback, and a waitlist control group, each group including 56 participants. Participants in the two iCBT conditions will undergo a 14-week treatment regimen, while those in the waitlist control group will wait for 14 weeks before starting iCBT with planned feedback. Additionally, participants in the iCBT groups will be randomly assigned to receive a booster session or not. The study design is factorial including two factors: type of therapist feedback (factor 1) and booster or no booster (factor 2). The study population comprises adolescents aged between 12 and 17 years, residing in Denmark, diagnosed with an anxiety disorder according to DSM-5 criteria. The primary outcome measures are the Youth Online Diagnostic Assessment and the Spence Children’s Anxiety Scale. Assessments will occur at baseline, post-treatment, and at 3-, 6-, and 12-month follow-ups post-treatment.

**Discussion:**

The findings of this study are anticipated to contribute to improving the accessibility of evidence-based treatments for adolescents with anxiety.

**Trial registration:**

The study is registered at clinicalTrials.gov, under protocol ID 22/59602. The Initial release was the 16.10.2023, first posted due to technical problems 16.04.2024. https://clinicaltrials.gov/study/NCT06368557?locStr=Odense,%20Denmark&country=Denmark&city=Odense&page=2&rank=13.

**Supplementary Information:**

The online version contains supplementary material available at 10.1186/s13063-024-08511-0.

## Administrative information

Note: the numbers in curly brackets in this protocol refer to SPIRIT checklist item numbers. The order of the items has been modified to group similar items (see http://www.equator-network.org/reporting-guidelines/spirit-2013-statement-defining-standard-protocol-items-for-clinical-trials/).

**Table:** 

Title **{**1}	Internet-based cognitive behavioral intervention for adolescents with anxiety disorders: a study protocol for a randomized controlled trial
Trial registration {2a} and {2b.}	ClinicalTrials.gov. ID: 22/59602 Internet-based Cognitive Behavioral Intervention for Adolescents With Anxiety Disorders
Protocol version {3}	10.06.2024, 1. version
Funding {4}	The study is funded by a grant from the Mental Health Services of Southern Denmark for the development of a state-of-the-art iCBT program and the investigation of the effectiveness of the program. The grant consists of 2.5 million DKK each year during the study. The grant is part of Center for Digital Psychiatry framework grant, and is allocated as budget.
Author details {5a}	1. Research Unit for Digital Psychiatry, Center for Digital Psychiatry, Mental Health Services in the Region of Southern Denmark, Odense, Denmark 2. Department of Clinical Research, Faculty of Health Sciences, University of Southern Denmark, Odense C, Denmark 3. Department of Psychology and Behavioral Sciences, School of Business and Social Sciences, Aarhus University, Aarhus, Denmark 4. Center for Emotional Health, Department of Psychology, Macquarie University, New South Wales, North Ryde, Australia HS:1,2 NMS:3 AHW:1 JJL:3 MT:3 LM:4 KM:1,2
Name and contact information for the trial sponsor {5b}	Center for Digital Psychiatry, Region of Southern Denmark Address: Heden 11, 5000 Odense C Phone number: 0045 99 44 95 50
Role of sponsor {5c}	Sponsor and funders have not had a role in the study design; collection, management, analysis and interpretation of data, writing of the report, and the decision to submit the report for publication, they will not have ultimate authority over any of these activities.

## Introduction

### Background and rationale {6a}

Anxiety disorders are the most prevalent mental health issues among children and adolescents [1, 2], affecting approximately 5–12% of youth in western countries [3, 4]. Research indicates a significant increase in anxiety disorder prevalence during the transition from childhood to adolescence [5]. Left untreated, anxiety disorders frequently become chronic or recurring, persisting into adulthood [6, 7].

Cognitive behavioral therapy (CBT) is an effective treatment for anxiety in young individual [8], recommended as the primary treatment option according to NICE guidelines [9]. Despite the availability of effective treatments and the potential long-term consequences of untreated anxiety disorders, less than 25% of affected youth seek professional help [10, 11] with even fewer receiving evidence-based treatment [12].

Common barriers preventing adolescents from seeking treatment include social stigma, shyness and fear of peer rejection [13–17], as well as preference for self-reliance [13, 17, 18]. Additionally concerns about confidentiality, privacy and anonymity [13, 14, 19], worries concerning treatment costs, transportation or waiting times [14, 20], and limited access to psychological services [13, 14, 21] are common barriers. Addressing these barriers is crucial for developing interventions that will enhance treatment accessibility.

Internet-based cognitive behavioral therapy (iCBT) presents a promising alternative to traditional face-to-face treatment, offering greater flexibility, autonomy, cost-effectiveness, and convenience [22]. Several randomized controlled trials (RCTs) have investigated the efficacy of iCBT programs for children and adolescents with anxiety disorders with promising results, including significant reductions in symptom severity [23–36]. Furthermore, a recent meta-analysis showed that Internet-based treatment is effective in reducing anxiety in youth when compared to both active and inactive control groups [37]. This trial seeks to examine the effectiveness of a newly developed iCBT program, CoolMinds, with the aim of facilitating its integration into routine care for adolescents in Denmark.

### Objectives {7}

The primary aim of the study is to examine the effectiveness of CoolMinds, an Internet-delivered cognitive behavioral therapy intervention, among adolescents aged 12–17 years. Specifically, the study seeks to examine the effectiveness of CoolMinds when administered with either planned feedback or on-demand feedback from a therapist, compared to a waitlist control group. Furthermore, the study will explore the effectiveness of delivering one booster session compared to no booster session in enhancing treatment outcomes.

We hypothesize that a higher proportion of participants in the two treatment conditions will achieve recovery from their primary anxiety diagnosis compared to those in the waitlist control group. Additionally, both treatment conditions are hypothesized to result in greater reduction in anxiety symptoms compared to the waitlist control group. We also hypothesize that the booster session will increase and better maintain reduction in anxiety symptoms over time.

### Trial design {8}

**Fig. 1:**
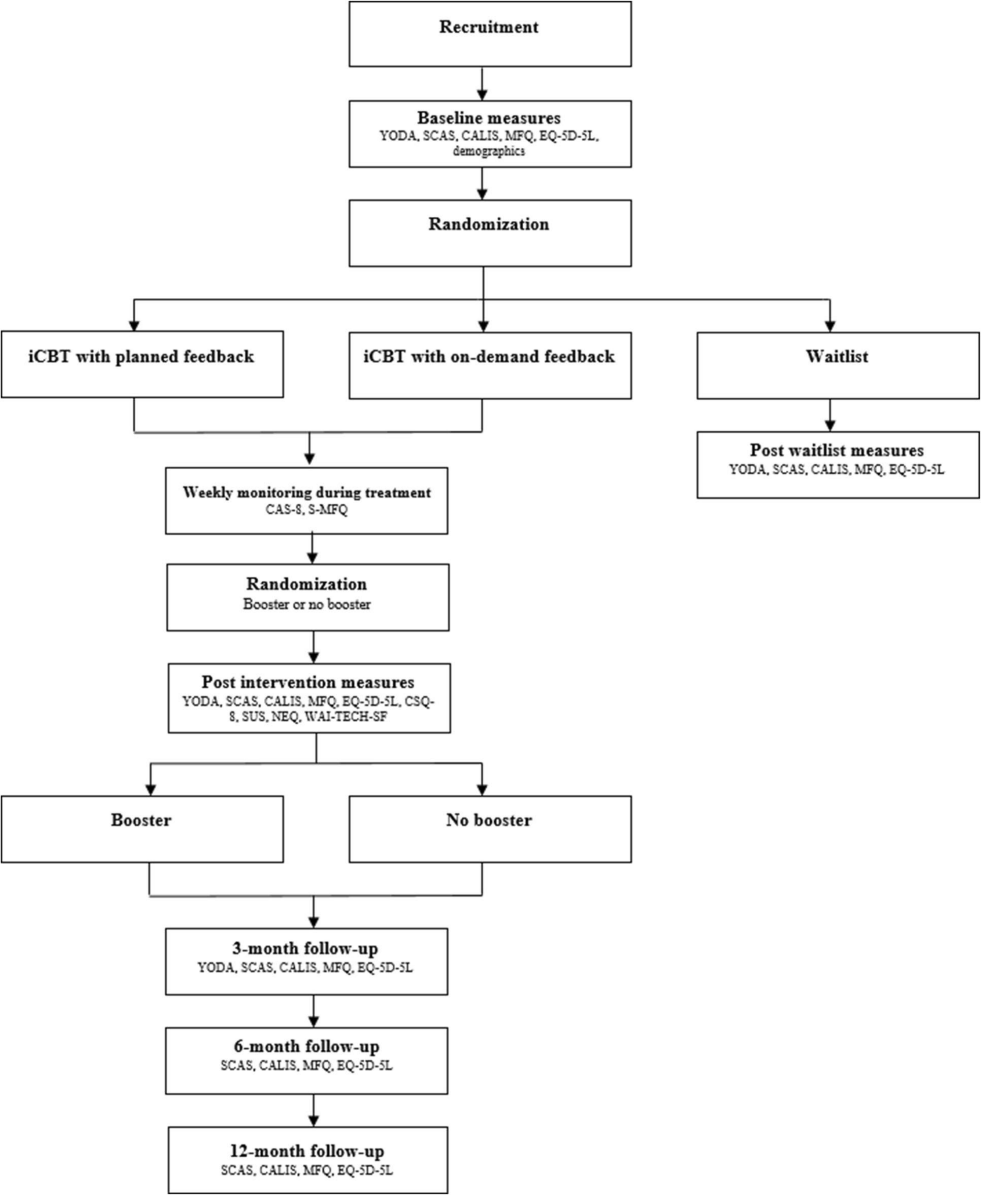
Participant flow diagram

The study is a randomized controlled trial (RCT) designed as a superiority trial with three parallel conditions: (1) iCBT with planned feedback, (2) iCBT with on-demand feedback and (3) waitlist control. The allocation ratio is 1:1:1 for each condition. The participants will be stratified by age into age groups 12–14 years and 15–17 years respectively to secure an even age distribution across conditions. Additionally, all active participants will be randomized to receive a booster session 10 weeks after end of treatment. The randomization will take part 10 weeks into treatment with an allocation ratio of 1:1. The design of the randomized trial is thus factorial including two factors: type of therapist feedback (factor 1) and booster or no booster (factor 2). Data will be collected using parent and adolescent questionnaires at five time points: pre-treatment, post-treatment and at follow-ups after 3, 6 and 12 months post-treatment. Participants in the waitlist condition will no longer take part in the study after the post waitlist measures, but will be offered iCBT with planned feedback after completing the waitlist period (Fig. 1).

## Methods: participants, interventions and outcomes

### Study setting {9}

The study will be conducted at the Centre for Digital Psychiatry (CEDIP), an online clinic and research facility at the psychiatric hospital in the Region of Southern Denmark, as well as Center for Psychological Treatment of Children and Adolescents (CEBU), a research and teaching center at Aarhus university, Denmark. All data will be collected from these two centers in Denmark.

### Eligibility criteria {10}

*Inclusion criteria:* Participants must be (1) between 12 and 17 years of age (both included) and have (2) a principal anxiety diagnosis according to DSM-5 criteria, as assessed by a clinical psychologist, (3) the ability to read and write Danish, (4) Internet access, and (5) a parent (or other legal guardian) able to participate in treatment alongside the adolescent. *Exclusion criteria:* (1) diagnosed with an autism spectrum disorder, (2) an Attention Deficit Hyperactive Disorder (ADHD/ADD), (3) psychotic symptoms, (4) bipolar disorder, (5) current suicidal ideation or self-mutilating behavior, (6) current alcohol or substance abuse, (7) a moderate to severe depression (8) current eating disorder and (9) received CBT for an anxiety disorder within the past 12 months.

The eligibility criteria for clinicians performing the intervention are as follows: must have a master’s degree in clinical psychology, and be employed at either Centre for Digital Psychiatry or Center for Psychological Treatment of Children and Adolescents.

### Who will take informed consent? {26a}

The research team will obtain online and oral informed consent from both the legal guardians and the participants between 15 and 17 years. If participants reach the age of majority (18 years old) during the trial, independent consent will be obtained. For participants between 12 and 14 years, online and oral informed consent will be obtained from their legal guardians.

All potential participants will be invited to a mandatory video call, where they will be informed about the study. Along with the invitation, will they receive written participant information, which they are encouraged to read before the call. During the video call, participants will have the opportunity to provide oral consent, or take time to reflect. If they choose to reflect, they can give oral consent by contacting the research staff within 1 week form the call. Once oral consent is obtained, participants will be asked to complete an online consent form. This consent will be collected using REDCap, a secure web platform for building and managing online databases and surveys [38, 39].

## Interventions

### Explanation for the choice of comparators {6b}

The use of a waitlist control is deemed necessary to investigate the effectiveness of the iCBT treatment compared to no treatment. In some of the most recent RCTs of iCBT for adolescents with anxiety disorders utilizing a waitlist control group, participants in this group also experienced an improvement in anxiety symptoms despite not receiving the intervention immediately [30, 32, 35].

Ambiguous results regarding the effect of dose and type of clinical support during iCBT treatment, coupled with a significant proportion of adolescents not fully responding to treatment, underscore the necessity for further research.

Research on adult iCBT programs suggests that even minimal therapist contact is sufficient to establish an adequate alliance [40, 41]. Interventions with lower degrees of guidance, such as automated support or on-demand support, have produced effect sizes comparable to interventions with higher levels of therapist guidance [42]. Similarly, within research on adolescent iCBT programs, the therapeutic alliance is evaluated as good even in iCBT formats with little to no therapist support [43]. Berg, Rozental [23] found no significant differences in treatment outcomes when iCBT was delivered with weekly feedback or weekly feedback with additional chat sessions. This suggests that only a limited amount of therapist support may be sufficient to obtain treatment effect, mirroring results from the research on adults.

### Intervention description {11a}

The Internet-based program, CoolMinds, is based on cognitive behavioral therapy for adolescents following the work by Kendall [44] and further developed by, e.g., Rapee, Lyneham [45] and Cunningham, Rapee [22]. The iCBT is delivered through online treatment modules, allowing adolescents to progress through the program at their own pace. The main treatment components include psychoeducation, cognitive restructuring, exposure therapy, and relapse prevention. The program comprises eleven sessions for adolescents and ten sessions for parents to be completed simultaneously over a 14-week period, including a booster session to be completed 10 to 12 weeks after end of treatment. The adolescent program and the parent program do not interact and are separate programs. See Tables 1 and 2 for an overview of the program content.

**Table 1:** Adolescent program content

Session		Content
1	Introduction	Welcome to the program. How to use the program and its functions.
2	Psychoeducation (specific)	Completing self-report measures on anxiety to obtain baseline functioning. Psychoeducation about specific anxiety disorders of choice: *social anxiety disorder, separation anxiety disorder, generalized anxiety disorder, panic disorder, specific phobias, OCD.*
3	Psychoeducation (general)	What is anxiety and what causes it? How many people experience anxiety?
4	Realistic thinking	Identification of problem situations. Identifying and challenging unrealistic thoughts.
5	Goalsetting and rewards	Setting goals and working towards them. Using rewards to maintain motivation.
6	Exposure 1	Creating an exposure hierarchy. Planning and executing exposure tasks. Reducing safety behavior.
7	Exposure 2	Creating an exposure hierarchy. Planning and executing exposure tasks. Reducing safety behavior.
7a	Exposure for OCD	Disorder specific content regarding exposure tasks and safety behavior.
7b	Exposure for panic disorder	Disorder specific content regarding exposure tasks and safety behavior.
8	Behavioral experiments	Planning and executing behavioral experiments and more challenging experiments.
9	The toolbox	Specific strategies to use when experiencing anxiety or difficulties with exposure, e.g., calm breathing, problem solving, constructive feedback.
10	Relapse prevention	Skills overview and maintenance. The journey of overcoming anxiety. Self-assessment of current challenges. What do I do if my anxiety returns or if I need more help?
11	What now?	Reward for completion. How to stay motivated and continue work.
12	Booster	Refreshing treatment techniques.

**Table 2:** Parent program content

Session		Content
1	Introduction	Welcome to the program. How to use the program and its functions.
2	Psychoeducation	What is my adolescent going through? Psychoeducation about specific anxiety disorders of choice: *social anxiety disorder, separation anxiety disorder, generalized anxiety disorder, panic disorder, specific phobias, OCD.* What is anxiety and what causes it? How many people experience anxiety?
3	Parent traps	Parental behavior. How anxious am I?
4	Do it yourself! Realistic thoughts	What will my child work with during the program? Trying out realistic thinking yourself (e.g., heights, water, spiders).
5	Do it yourself! Goals and rewards	What will my child work with during the program? Trying out goalsetting and rewards yourself.
6	Do it yourself! Exposure	What will my child work with during the program? Trying out exposure tasks yourself (e.g., heights, water, spiders).
7	School	How to handle school absenteeism due to anxiety. Helping the adolescent cope with anxiety in school. Includes an informal print-out to be handed out to, e.g., teachers.
8	Relapse prevention	The journey of overcoming anxiety. How to support the adolescent during difficulties.
9	Need more help	Where can I seek more help for my own potential difficulties? Where can I as a parent seek more help for my adolescent?
10	What now?	How to continue supporting the adolescent.
11	Booster	Refreshing treatment techniques.

#### iCBT with planned feedback

Participants in the planned feedback iCBT condition will receive written feedback from their therapist on assignments every week. They will also have the opportunity to contact their assigned therapist through messages via a built-in text module within the program and receive asynchronous support if needed.

#### iCBT with on-demand feedback

Participants in the on-demand feedback iCBT condition will not receive any planned contact with the therapist. However, they will have the option to contact the therapist through messages within the program and receive asynchronous support.

In both treatment conditions, the therapist may spend a maximum of 15 min giving feedback per week. While we have advised therapists to adhere to this standard time used per participant in The Internet psychiatry, we cannot fully ensure adherence to this rule as it is up to the discretion of each therapist. Since there is no evidence on what amount of therapist support is sufficient for adolescents, the allowed time spent giving feedback will not differ between the two conditions. Participant depression and suicidality will also be continuously monitored in both intervention conditions using in-session questionnaires (see section “Plans for assessment and collection of outcomes {18a}”).

#### Waitlist

Participants in the waitlist condition will be instructed to wait for 14 weeks. After this waiting period, they will no longer take part in the study, but will be offered iCBT treatment with planned therapist feedback. If participants do not wish to receive the iCBT treatment, they will receive assistance in finding another relevant treatment if needed.

### Criteria for discontinuing or modifying allocated interventions {11b}

If an adolescent shows an increase in co-morbid depressive symptoms above the cutoff for clinical depression, or if they show signs of suicidal ideation, the clinician will contact both the participant and their legal guardians to discuss whether the participant should be discontinued from the study and receive help finding other treatment if needed. The clinician may contact the participants up to three times during the trial to investigate depression or suicidal ideation. If the participant shows increase in depressive symptoms or exhibits suicidal ideation for the third time during the treatment, the purpose of the contacts will be to discontinue the participant from the study and help them find other treatment if needed.

### Strategies to improve adherence to interventions {11c}

To improve adherence to the intervention, the therapist will actively monitor the adolescent’s engagement with the treatment program. If an adolescent fails to engage with the program for 10 consecutive days, both the adolescent and the parents will be given the option of a motivational video call. This call will serve to explore the reasons behind the lack of participation and to assist in developing strategies to help the adolescent continue with the treatment. The motivational video call should not include any form of CBT, and will only be offered once per family.

### Relevant concomitant care permitted or prohibited during the trial {11d}

Participants are not allowed to partake in other psychotherapeutic anxiety treatments during participation, unless it is deemed unethical to prohibit such treatment, which will be assessed on a case-by-case basis. This assessment will occur during the informational video call, as well as in pre- and post-treatment questionnaires. Participants who mention seeking additional care for anxiety during the video call will be advised to only partake in one treatment at the time, unless it is deemed unethical for the participants. There are no restrictions on seeking additional non-anxiety-related care, but data on additional care utilized during the treatment period will be obtained at post-treatment.

Stable medication during participation is preferred, but we do not have the authority to administer or regulate medication, so any changes in medicine during the treatment period will also be obtained at post-treatment. Since the RCT follows the intention to treat principle, participants who receive additional treatment or medication will not be excluded from the statistical analysis. However, the effect of additional treatment and medication will be discussed in the article.

### Provisions for post-trial care {30}

If any participants suffer a serious negative effect of treatment resulting in psychological, physical, or financial loss, the research team is obliged to inform the participants about the Danish Patient Compensation scheme. If necessary, the team will also assist the participants in applying for compensation.

### Outcomes {12}

#### Primary outcome

The primary outcome consists of point differences in anxiety diagnosis and severity measured by Spence Children’s Anxiety Scale (SCAS) [46]. SCAS is a 44-item self-report questionnaire assessing anxiety symptoms of six different anxiety disorders in DSM-IV. SCAS has been validated in a Danish community and clinical sample of children between the age of 7 and 17 years and the results indicated good internal consistency, test-retest reliability and convergent and divergent validity for the questionnaire [47].

#### Secondary outcomes

The secondary outcomes consists of point differences in anxiety diagnosis and severity measured by clinician-review Youth Online Diagnostic Assessment-Child and Parent Versions (YODA) [48]. YODA is an online diagnostic assessment tool that assesses DSM-5 anxiety disorders and specific phobias based on the *Anxiety and Related Disorders Interview Schedule for DSM-5*, which is considered the golden standard [49]. The YODA uses yes/no responses to closed-ended questions, as well as open-ended questions that require written descriptions of cognitions, behaviors and impact on functioning [48]. The YODA is completed separately by the adolescent and the parents, and a clinical psychologist trained in the assessment of adolescent anxiety disorders then reviews and evaluates the responses to determine the diagnosis and symptom severity.

Point differences in anxiety symptoms interference on daily life measured by the self- and parent-reported questionnaire Child Anxiety Life Interference Scale (CALIS) [46]. CALIS [50] is used to measure the impact of youth anxiety on various areas of life functioning such as school, extracurricular activities, family life and friendships. The impact is evaluated separately by adolescents and their parents. In addition, parents rate the degree of impact on their own lives. CALIS has shown satisfactory internal consistency and moderate test-retest reliability [50].

Point differences in depressive symptoms are measured by The mood and Feelings Questionnaire (MFQ) [51]. MFQ is used to screen for depression in youth, both in epidemiological samples and in clinical samples, it is recommended as a self-report screening tool in secondary care [51]. The MFQ is completed separately by the adolescent and a parent. The MFQ has been validated in a Danish population sample of adolescents and shown excellent internal consistency [52].

Point differences in Quality of life is measured by EuroQol-5 Dimension Youth (EQ-5D-5L) [52], a 5-item self-report questionnaire assessing quality of life and self-rated health. The items cover five domains: mobility, self-care, usual activities, pain/discomfort, and anxiety and depression [53].

Other secondary outcomes include outcomes related to overall participant experience, they are therefore not administered at pre-treatment. This includes participant satisfaction with treatment and with system usability, measured by Working Alliance Inventory applied to Internet (WAI-TECH-SF) [54]. WAI-TECH-SF assesses the technical alliance. The WAI-TECH-SF has shown good psychometric properties and excellent internal consistency [54].

Working Alliance Inventory – Short Form (WAI-SF) [55]. The therapeutic alliance will be assessed using the short version of the Working Alliance Inventory. The WAI-SF is a 12-item self-report questionnaire designed to assess the therapeutic alliance with the therapist / online program in a self-guided intervention on three dimensions: therapeutic goals, tasks, and bonds. The WAI-SF will be administered after week 2, 4, 6, 8 and 10 and at post-treatment and be completed by both adolescents and parents.

Client Satisfaction Questionnaire-8 (CSQ-8) [56] is used to measure general satisfaction with the received treatment. The scale has shown high internal consistency and concurrent validity [57].

System Usability scale (SUS) [58] is used to assess the subjective experience of usability of a computer system. A Danish version of the SUS has been validated in a mental health care setting [59].

Negative Effect Questionnaire (NEQ) [60] is a 20-item questionnaire used to monitor the occurrence of negative effects in psychological treatments. NEQ shows acceptable psychometric properties [60].

Engagement with the treatment program will be measured objectively using the number of logins to the treatment program as well as number of words per message sent to the therapist and number of words per text box in the modules. These data are automatically logged in the treatment platform and will be registered for both adolescents and parents.

Completion of the treatment program is defined as completing module 8 for adolescents or being active in the treatment program for all 14 weeks.

#### Weekly outcomes

The 8-item Children’s Anxiety Scale (CAS-8) [61]. The CAS-8 is a short version of the SCAS containing 8 items. I has shown acceptable psychometric properties, with good reliability [61].

The Short Mood and Feelings questionnaire (S-MFQ) [62]. The S-MFQ is a short version of MFQ, at shows high accuracy for discriminating MDD cases from non-cases [63].

Sociodemographic measures will be gathered on parents regarding age, level of education, job status, civil status, gender, and primary caregiver in case of single parents. For the adolescents, information will be gathered regarding age and gender.

### Participant timeline {13}

All participants will need to register through a website to participate in the project. During registration, participants will be required to complete an initial screening questionnaire to assess exclusion criteria. If an exclusion criterion is met, the participant will automatically be informed within the questionnaire that they are unable to participate and will be given the choice to either continue or discontinue their application process.

Participants who complete the screening questionnaire and who are not excluded are invited to participate in a video call with one of the research staff members, during which they will receive information about the study, their rights, and a consent form. Participants will have the option to verbally consent to participation during the video call, or they may be prompted to schedule another time to clarify their participation.

Participants who provide both written and verbal consent will receive an online questionnaire package. For adolescents, this package will include the following assessments: The Youth Online Diagnostic Assessment (YODA), Spence Children’s Anxiety Scale (SCAS), Child Anxiety Life Interference Scale (CALIS), The Mood and Feelings Questionnaire (MFQ), and EuroQol-5 Dimension Youth (EQ-5D-5L). For parents, the package will include the caregiver version of YODA, SCAS-C/P, MFQ, and CALIS.

After completing the questionnaires, participants will be invited to a video call with a clinical psychologist for an assessment interview. Based on the collected data and the assessment interview, the clinical psychologist will determine if the participant meets the diagnostic criteria for an anxiety diagnosis according to DSM-5, and are eligible to take part in the study. If eligible, the participant will be randomized in to the three conditions. If the participants are in the two treatment conditions they will be provided with login credentials for the treatment platform. If the participants are in the waitlist, the will be notified that they will get the login credentials after answering the post-waitlist questionnaires.

Participants and their parents will engage in the treatment for 14 weeks. They will have access to the platform for an additional 12 weeks after end of the treatment period. After 10 weeks of active participation in the treatment, the participants in the intervention conditions will be randomized to either receive a post-treatment booster session or not. Participants allocated to the booster condition will gain access to the booster module 24 weeks after receiving their login.

Immediately after the 14-week treatment period, participants will be assessed with post-treatment questionnaires and invited to a final video call with the clinical psychologist to conclude the active part of treatment.

Follow-up measurements will be conducted 3, 6, and 12 months after completing the treatment (Table 3).

**Table 3:**
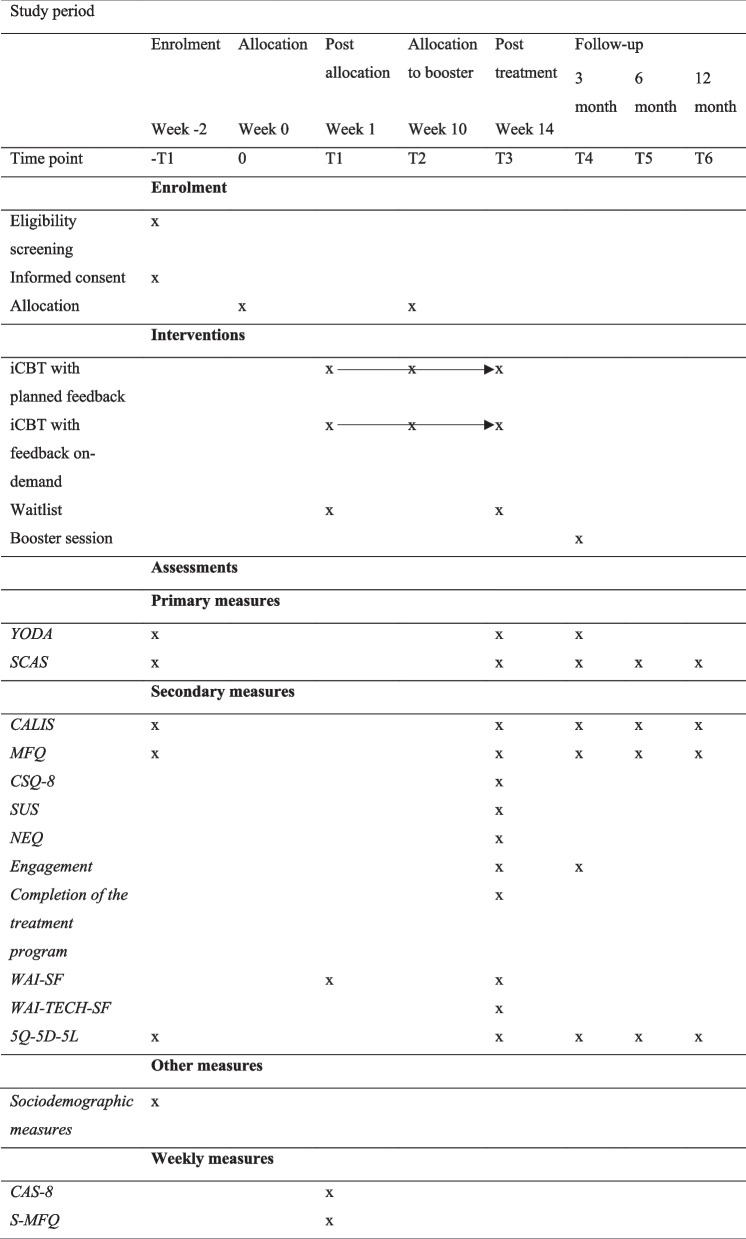
Participant timeline

### Sample size {14}

The sample size calculation is based on scenarios of difference between treatment and waitlist at end of treatment for the primary outcome SCAS, based on Stjerneklar and colleagues [32] previous study detecting a difference of 10 points between the waiting list and the (combined) treatment groups in change in SCAS score from baseline to 3 months’ follow-up. Using a linear mixed model with outcome variance *σ*^2^=225 (*σ* = 15) and intraclass coefficient ICC=0.6 [32], and with significance level *a* = 0.05 and power = 1−b = 0.80, allowing for unequal group sample sizes (randomized 1:2), a total sample size of 134 patients is needed [64, 65]. Allowing for 20% attrition, a total of *n* = 168 patients (*n* = 56 waiting list, *n* = 112 treatment) will be included.

### Recruitment {15}

Participants will be recruited through self-referral and referral from health specialists within Denmark. The project will be promoted on Facebook and Instagram.

If social media advertisement is not sufficient, information letters will be sent to educational psychologists, general practitioners (GPs), and adolescent mental health care units. Additionally, letters will be sent to counseling services operated by volunteers and non-governmental organizations.

The recruitment will proceed until 168 participants are enrolled or until all clinicians are fully booked. If all clinicians are fully booked, recruitment will be temporarily paused until additional clinician availability becomes available.

## Assignment of interventions: allocation

### Sequence generation {16a}

The trial will use an allocation ratio of 1:1:1. Participants will be stratified by age in two categories 12–14 years of age and 15–17 respectively. Block randomization will be used with variable block sizes. To reduce predictability of a random sequence, the block sizes are made with random block sizes in Stata version 18 [66] by a statistician, and neither the research team, the clinicians or the administrative team have access to this information at any point of the trial.

### Concealment mechanism {16b}

The random block sizes are concealed in REDCap [38, 39] by our data manager. There is not possible for the research team, the clinicians, or the administrative team to see the allocation of the participant before the intervention is assigned. The intervention is assigned using the randomization function in REDCap, it is not possible to change allocation for the participant at any time of the study as this function in REDCap is blocked by our data manager.

### Implementation {16c}

A statistician will generate the allocation sequence, the clinicians will enroll participants, and assign participants to the interventions.

## Assignment of interventions: Blinding

### Who will be blinded {17a}

Given the nature of the study examining the effectiveness of a novel format of psychotherapy and the examination of difference in therapist support, blinding participants and clinicians is not possible. Additionally, the research team actively engages in day-to-day project operations, including the screening for suicidality, and therefore, the research team is not blinded.

However, to mitigate bias, the trial results will be analyzed using pseudo-anonymized data. This means that only the ID numbers will appear on the data sheet, aiming to achieve as close to assessor blinding as possible within the research design.

## Data collection and management

### Plans for assessment and collection of outcomes {18a}

All questionnaire measures will be collected using REDCap [38, 39]. Participants will receive automatic reminders sent after 4 and 8 days. If participants have not completed the questionnaires after receiving reminders, a member of the research team will contact them by phone in an attempt to re-engage them.

For post-treatment and follow-up measures, contact will be attempted a maximum of 3 times in total at different time points to prompt completion.

### Plans to promote participant retention and complete follow-up {18b}

If a participant is inactive (e.g., did not log in to the platform) for more than 10 days, the therapist will initially contact the participant through the chat function on the platform. The purpose of this contact is to assess the reasons for inactivity and to schedule a motivational conversation with the participant and their parents if necessary.

If the participant does not respond to the chat messages within 2 days, the therapist will contact them by telephone to assess the reasons for inactivity and schedule a motivational conversation. These conversations may last up to 45 min and aim to help the participant identify reasons for change and address potential treatment barriers.

The participant will be encouraged to continue working with the program during these conversations. However, if inactivity persists, the participant will be discontinued from the treatment and assisted in finding a more suitable treatment option.

All participants enrolled in the study will receive the post- and follow-up measures, unless they specifically decline participation in these. Therefore, even participants who do not complete the treatment will still be assessed with all measures.

### Data management {19}

The data will be collected in REDCap and MindDistrict, the data will be stored in the REDCap database or on SharePoint during and after the trial. Every 6 months, a data control will be carried out to make sure the data appears as expected. In REDcap, there are inbuilt validations for the questionnaires, and all questions are required to be answered to complete the questionnaires.

### Confidentiality {27}

Any personally sensitive information is processed by Center for Digital Psychiatry and Aarhus University in personally identifiable form for as long as it is necessary for the research purpose. The storage of information complies with responsible research practice and general data protection regulation. Once the information is no longer necessary for the research project, it will be anonymized or deleted. During this trial, no personally sensitive information will be shared, and all data will be anonymized before publishing the results.

## Statistical methods

### Statistical methods for primary and secondary outcomes {20a}

The primary analysis are the association between the change in SCAS-C/P total score from pre- to post-treatment and trial condition will be analyzed using linear mixed model regression.

For the secondary analysis, the association between YODA, dichotomized as YODA total score ≥ 1 vs. YODA total score < 1, and the three trial conditions at post-treatment will be analyzed using logistic regression.

A more detailed analysis plan will be developed prior to the end of recruitment for both primary and secondary analyses in collaboration with a statistician employed at the participating organizations.

### Interim analyses {21b}

The data monitoring committee will conduct monthly analyses to ensure that there are no significant harms related to participating in this project. The members of the data monitoring committee will all have access to these interim analyses. Termination of the trial will be decided in unison, but in case of disagreement, the final decision lies with the primary investigator.

### Methods for additional analyses (e.g., subgroup analyses) {20b}

A large number of additional analyses will be conducted. Logistic regression will be used to analyze associations between treatment arms and the secondary outcome YODA (dichotomized) at 3-month follow-up post treatment as well as each of the 20 NEQ items at post-treatment. Linear mixed model regression will be used to analyze associations between treatment arms and the following outcomes through all time points from pre-treatment to 12-month follow-up post treatment: SCAS-C score, CALIS, MFQ score by adolescent, SCAS-P, CALIS, MFQ score by parent, EQ-5D-5L score, CSQ-8 score, and SUS score. Furthermore, linear mixed model regression will be used to analyze associations between treatment arms and WAI-SF at treatment sessions 2, 4, 6, 8, and 10 and at post treatment, and between treatment arms and weekly measures of CAS-8 and S-MFQ. Linear regression will be used to analyze associations between treatment arms and WAI-TECH-SF and engagement at post-treatment, as well as the cost-effectiveness analysis.

Finally, we wish to conduct a post hoc exploratory growth mixture model that can provide trajectories of change in symptoms through the trial across the different sessions and post-measurements.

### Methods in analysis to handle protocol non-adherence and any statistical methods to handle missing data {20c}

Linear mixed models, which tolerate missing values and minimize the loss of statistical power in case of drop-out, will be fitted using the maximum-likelihood estimation method and based on the intention-to-treat sample. Where relevant in other types of analyses, missing data will be handled using multiple imputation methods.

### Plans to give access to the full protocol, participant-level data, and statistical code {31c}

Data will be available for researches at the Center for Psychological Treatment of Children and Adolescents and researches from the Centre for Digital Psychiatry. They will have access to the study protocol, the statistical analysis plan, informed consent form, and analytical code.

Data will be stored on a server located in the Region of Southern Denmark. When the study is completed, the data will be transferred to The Danish National Archives. Data is available upon reasonable request. Restrictions apply to the availability of data and approval is needed from Danish Data Protection Agency and/or The Danish National Archives.

## Oversight and monitoring

### Composition of the coordinating center and trial steering committee {5d}

CEDIP is responsible for recruiting participants, delivering the program, and managing the database and research data as described in this protocol.

The research team will work as the trial steering committee, comprising members from both CEBU and CEDIP. They will hold meetings every third week for 1 h to oversee the trial’s day-to-day operations, monitor progress, and provide supervision.

The data managing team consists of one data manager from Open Patient Data Explorative Network (OPEN) collaborating with one data manager from CEDIP. The data managing team is in continuous contact with the research team.

### Composition of the data monitoring committee, its role and reporting structure {21a}

A data monitoring committee will be established, consisting of clinicians working on the project, the project data manager, and two PhD students. The committee’s role is to ensure data quality and monitor potential negative effects of participation in the study.

It’s important to note that this committee is not independent from the sponsor, as this study is internally funded. However, the data manager, who is connected to another hospital unit, is tasked with ensuring proper data management across the hospital and thus has no competing interests in the project.

### Adverse event reporting and harms {22}

Another committee, comprising clinicians working on the project and members of the research team, will be established to monitor individual risks on a case-to-case basis. This committee will oversee screening for depression and suicidality using S-MFQ scores, and anxiety symptom severity using CAS-8. Screening will occur daily, with exceptions for holidays and weekends.

Additional negative effects will be assessed through the NEQ after the adolescent have completed 14 weeks of treatment. If any risks or harms are detected, the committee will report to the assigned clinician. It will then be the clinician’s responsibility to contact the participant, and/or their legal guardians to investigate if the participant needs additional help. All adverse events will be recorded in the trials database.

### Frequency and plans for auditing trial conduct {23}

The project will be closely monitored by the Principal clinical investigator, who is internal to the project. Every 6 months, the Principal Clinical Investigator will conduct an inspection to ensure compliance with the study protocol and the guidelines for Good Clinical Practice [67].

To ensure adherence to the study protocol, all researchers involved in the project must commit to following a field guidebook with detailed instructions for each step of the study.

Additionally, the Regional Committees on Health Research Ethics for southern Denmark has the authority to inspect the project during the trial period to ensure compliance with the approved ethical protocol.

### Plans for communicating important protocol amendments to relevant parties (e.g., trial participants, ethical committees) {25}

Having completed a feasibility trial resulting in adaptations and amendments to the protocol approved by the ethical committee, we anticipate minimal further amendments. However, if significant amendments are necessary, we will promptly communicate them to the National Committees on Health Research Ethics of Denmark for approval.

Similarly, any important amendments that affect participant participation or their experience in the trial will be promptly communicated to participants, no later than 1 week after approval and implementation. In cases of major amendments, the participants will be contacted by phone by a member of the research team.

Additionally, information regarding amendment will be published on the CoolMinds.dk website. All relevant parties will also be informed by mail by the research team.

### Dissemination plans {31a}

The results of the project will be submitted to high-impact international and peer-reviewed journals under open access, and presented at national and international conferences. Additionally, the research team will organize public lectures, courses, and workshops to disseminate understanding of anxiety in youth and treatment options among professionals and the general public.

A layman summary of the results will be composed and forwarded to participants who have expressed interest, as well as published on the CoolMinds website. Currently, six scientific articles are planned to be submitted based on the results from the project, which will be part of two PhD dissertations.

The following six articles are planned:

- Paper 1: A systematic review of research on iCBT and the use of support and booster sessions
- Paper 2: Predictor analysis of treatment outcome
- Paper 3: Results from the feasibility trial
- Paper 4: Results from the RCT
- Paper 5: Trajectory of change multiple single case descriptions
- Paper 6: Network analysis of symptoms, severity, and change from pre- to post-treatment.

## Discussion

The present study aims to investigate the clinical effectiveness of the iCBT intervention CoolMinds when delivered with either planned therapist feedback or on-demand therapist feedback. Additionally, the study aims to examine the effects of providing one booster session 10 weeks after completion of the treatment program. The online format may help overcome barriers for adolescents seeking anxiety treatment without compromising the effectiveness of traditional CBT.

A strength of the study lies in the identical content of the CoolMinds intervention in both active conditions, allowing for a direct comparison of the therapist support levels in iCBT for adolescents. Furthermore, a feasibility trial has already been conducted, mitigating concerns regarding technical problems and operational issues. However, initial low recruitment numbers and potential high dropout rates may delay the study and challenge the examination of booster session effects. Additionally, the study’s reliance on a small number of clinicians leaves it vulnerable to long-term absences or discontinuation.

This study will contribute to the existing literature with knowledge on the effectiveness of iCBT in adolescents with anxiety when comparing therapist feedback levels and examining the effects of booster sessions. The results aim to improve the accessibility of evidence-based treatment for adolescents with anxiety.

## Trial status

Version number 1.

Recruitment began September 1st 2023. The first participants were included and started treatment on October 18th.

Recruitment is approximated to end June 2025.

## Supplementary Information

Supplementary Material 1.

## Data Availability

Data will be available for researches internal to the project at Aarhus University, Center for Psychological treatment of Children and Adolescents and researchers from Center for Digital Psychiatry. They will have access to the study protocol, statistical analysis plan, informed consent form, and analytical code.

## Abbreviations

CBT: Cognitive Behavioral Therapy
CEBU: Center for Psychological Treatment of Children and Adolescents
CEDIP: Center for Digital Psychiatry
DSM-V: Diagnostic and Statistical Manual of Mental Diseases V
iCBT: Internet-based cognitive behavioral therapy
ICC: Intraclass coefficient
GP: General Practitioner
NGO: Non-governmental organization
OCD: Obsessive compulsive disorder
OPEN: Open Patient data Explorative Network
RCT: Randomized controlled trial
SCAS: Spence Child Anxiety Scale
YODA: Youth Online Diagnostic Assessment
S-MFQ: The Short Mood and Feelings questionnaire
CAS-8: The 8-item Children’s Anxiety Scale
DSM-5: Diagnostic and Statistical Manual of Mental Disorders
ADHD: Attention deficit hyperactivity disorder
ADD: Attention deficit disorder
CALIS-C: Child Anxiety Life Interference Scale-Children
CALIS-P: Child Anxiety Life Interference Scale-Parent
MFQ-C: The Mood and Feelings Questionnaire-Children
MFQ-P: The Mood and Feelings Questionnaire-Parent
EQ-5D-5L: EuroQol-5 Dimension Youth
NEQ: Negative Effects Questionnaire
SUS: Systems Usability Scale
WAI-TECH-SF: Working Alliance Inventory applied to Internet
WAI-SF: Working Alliance Inventory – Short Form
ICC: Intraclass correlation coefficient
ID: Identity

## References

[1] Costello EJ, Egger HL, Copeland W, Erkanli A, Angold A. The developmental epidemiology of anxiety disorders: phenomenology, prevalence, and comorbidity. In: Silverman WK, Field AP, editors. Anxiety disorders in children and adolescents. Cambridge: Cambridge University Press; 2011. p. 56–75.

[2] Kessler RC, Berglund P, Demler O, Jin R, Merikangas KR, Walters EE. Lifetime prevalence and age-of-onset distributions of DSM-IV disorders in the national comorbidity survey replication. Arch Gen Psychiatry. 2005;62(6):593–602. 15939837 10.1001/archpsyc.62.6.593

[3] Merikangas KRPD, J-pMS He, Burstein MPD, Swanson SASM, Avenevoli SPD, Cui LMS, et al. Lifetime prevalence of mental disorders in U.S. adolescents: results from the national comorbidity survey replication-adolescent supplement (NCS-A). J Am Acad Child Adolesc Psychiatr. 2010;49(10):980–9. 10.1016/j.jaac.2010.05.017 PMC2946114 20855043

[4] Polanczyk GV, Salum GA, Sugaya LS, Caye A, Rohde LA. Annual research review: a meta-analysis of the worldwide prevalence of mental disorders in children and adolescents. J Child Psychol Psychiatry. 2015;56(3):345–65. 25649325 10.1111/jcpp.12381

[5] Beesdo K, Knappe S, Pine DS. Anxiety and anxiety disorders in children and adolescents: developmental issues and implications for DSM-V. Psychiatr Clin. 2009;32(3):483–524. 10.1016/j.psc.2009.06.002 PMC3018839 19716988

[6] Keller MB, Lavori PW, Wunder J, Beardslee WR, Schwartz CE, Roth J. Chronic course of anxiety disorders in children and adolescents. J Am Acad Child Adolesc Psychiatry. 1992;31(4):595–9. 1644719 10.1097/00004583-199207000-00003

[7] Kessler RC, Avenevoli S, Costello EJ, Georgiades K, Green JG, Gruber MJ, et al. Prevalence, persistence, and sociodemographic correlates of DSM-IV disorders in the national comorbidity survey replication adolescent supplement. Arch Gen Psychiatry. 2012;69(4):372–80. 22147808 10.1001/archgenpsychiatry.2011.160 PMC3445020

[8] Cartwright-Hatton S, Roberts C, Chitsabesan P, Fothergill C, Harrington R. Systematic review of the efficacy of cognitive behaviour therapies for childhood and adolescent anxiety disorders. Br J Clin Psychol. 2004;43(4):421–36. 15530212 10.1348/0144665042388928

[9] National Institute for Health and Care Excellence (NICE). Social anxiety disorder: recognition, assessment and treatment. National Clinical Guideline Number 159. Leicester (UK): The British Psychological Society and The Royal College of Psychiatrists; 2013. [cited 2003 12 20]. Available from: https://www.nice.org.uk/guidance/cg159/.

[10] Merikangas KRPD, He JPMS, Burstein MPD, Swendsen JPD, Avenevoli SPD, Case BMD, et al. Service utilization for lifetime mental disorders in U.S. adolescents: results of the National Comorbidity Survey–Adolescent supplement (NCS-A). J Am Acad Child Adolesc Psychiatry. 2011;50(1):32–45. 21156268 10.1016/j.jaac.2010.10.006 PMC4408275

[11] Wang PS, Angermeyer M, Borges G, Bruffaerts R, Tat Chiu W, De Girolamo G, et al. Delay and failure in treatment seeking after first onset of mental disorders in the World Health Organization’s world mental health survey Initiative. World Psychiatr. 2007;6(3):177–85. PMC2174579 18188443

[12] Reardon T, Harvey K, Creswell C. Seeking and accessing professional support for child anxiety in a community sample. Eur Child Adolesc Psychiatry. 2019;29(5):649–64. 31410579 10.1007/s00787-019-01388-4 PMC7250799

[13] Booth ML, Bernard D, Quine S, Kang MS, Usherwood T, Alperstein G, et al. Access to health care among Australian adolescents young people’s perspectives and their sociodemographic distribution. J Adolesc Health. 2004;34(1):97–103. 14706412 10.1016/j.jadohealth.2003.06.011

[14] Gulliver A, Griffiths KM, Christensen H. Perceived barriers and facilitators to mental health help-seeking in young people: a systematic review. BMC Psychiatry. 2010;10(1):113-. 21192795 10.1186/1471-244X-10-113 PMC3022639

[15] Jaycox LH, McCaffrey DF, Ocampo BW, Shelley GA, Blake SM, Peterson DJ, et al. Challenges in the evaluation and implementation of school-based prevention and intervention programs on sensitive topics. Am J Eval. 2006;27(3):320–36.

[16] Nearchou FA, Bird N, Costello A, Duggan S, Gilroy J, Long R, et al. Personal and perceived public mental-health stigma as predictors of help-seeking intentions in adolescents. J Adolesc. 2018;66(C):83–90. 29800758 10.1016/j.adolescence.2018.05.003

[17] Rickwood DJ, Deane FP, Wilson CJ. When and how do young people seek professional help for mental health problems? Med J Aust. 2007;187(S7):S35–9. 17908023 10.5694/j.1326-5377.2007.tb01334.x

[18] Rickwood DJ, Braithwaite VA. Social-psychological factors affecting help-seeking for emotional problems. Soc Sci Med. 1994;39(4):563–72. 7973856 10.1016/0277-9536(94)90099-x

[19] Musiat P, Goldstone P, Tarrier N. Understanding the acceptability of e-mental health-attitudes and expectations towards computerised self-help treatments for mental health problems. BMC Psychiatry. 2014;14(1):1–8. 10.1186/1471-244X-14-109 PMC3999507 24725765

[20] Stallard P, Richardson T, Velleman S. Clinicians’ attitudes towards the use of computerized cognitive behaviour therapy (cCBT) with children and adolescents. Behav Cogn Psychother. 2010;38(5):545–60. 20615273 10.1017/S1352465810000421

[21] Stallard P, Velleman S, Richardson T. Computer use and attitudes towards computerised therapy amongst young people and parents attending child and adolescent mental health services. Child Adolesc Mental Health. 2010;15(2):80–4. 10.1111/j.1475-3588.2009.00540.x 32847246

[22] Cunningham M, Rapee R, Lyneham H. The cool teens CD-ROM: a multimedia self-help program for adolescents with anxiety. Youth Stud Aust. 2006;25(1):50–6.

[23] Berg M, Rozental A, de Brun Mangs J, Näsman M, Strömberg K, Viberg L, et al. The role of learning support and chat-sessions in guided internet-based cognitive behavioral therapy for adolescents with anxiety: a factorial design study. Front Psychiatr. 2020;11:503. 10.3389/fpsyt.2020.00503 PMC7298729 32587533

[24] Jolstedt M, Wahlund T, Lenhard F, Ljótsson B, Mataix-Cols D, Nord M, et al. Efficacy and cost-effectiveness of therapist-guided internet cognitive behavioural therapy for paediatric anxiety disorders: a single-centre, single-blind, randomised controlled trial. Lancet Child Adolesc Health. 2018;2(11):792–801. 30241993 10.1016/S2352-4642(18)30275-X

[25] Khanna MS, Kendall PC. Computer-assisted cognitive behavioral therapy for child anxiety: results of a randomized clinical trial. J Consult Clin Psychol. 2010;78(5):737–45. 20873909 10.1037/a0019739

[26] Leigh E, Clark DM. Internet‐delivered therapist‐assisted cognitive therapy for adolescent social anxiety disorder (OSCA): a randomised controlled trial addressing preliminary efficacy and mechanisms of action. J Child Psychol Psychiatry. 2023;64(1):145–55 10.1111/jcpp.13680 PMC10087225 35943064

[27] March S, Spence SH, Donovan CL. The efficacy of an internet-based cognitive-behavioral therapy intervention for child anxiety disorders. J Pediatr Psychol. 2008;34(5):474–87. 18794187 10.1093/jpepsy/jsn099

[28] Nordh M, Wahlund T, Jolstedt M, Sahlin H, Bjureberg J, Ahlen J, et al. Therapist-guided internet-delivered cognitive behavioral therapy vs internet-delivered supportive therapy for children and adolescents with social anxiety disorder: a randomized clinical trial. JAMA Psychiatry (Chicago, Ill). 2021;78(7):705–13. 10.1001/jamapsychiatry.2021.0469 PMC8117054 33978699

[29] Spence SH, Donovan CL, March S, Gamble A, Anderson RE, Prosser S, et al. A randomized controlled trial of online versus clinic-based CBT for adolescent anxiety. J Consult Clin Psychol. 2011;79(5):629–42. 21744945 10.1037/a0024512

[30] Spence SH, Donovan CL, March S, Kenardy JA, Hearn CS. Generic versus disorder specific cognitive behavior therapy for social anxiety disorder in youth: a randomized controlled trial using internet delivery. Behav Res Ther. 2017;90:41–57. 27988427 10.1016/j.brat.2016.12.003

[31] Spence SH, Holmes JM, March S, Lipp OV. The feasibility and outcome of clinic plus internet delivery of cognitive-behavior therapy for childhood anxiety. J Consult Clin Psychol. 2006;74(3):614. 16822117 10.1037/0022-006X.74.3.614

[32] Stjerneklar S, Hougaard E, McLellan LF, Thastum M. A randomized controlled trial examining the efficacy of an internet-based cognitive behavioral therapy program for adolescents with anxiety disorders. PloS One. 2019;14(9):e0222485-e. 31532802 10.1371/journal.pone.0222485 PMC6750608

[33] Storch EA, Salloum A, King MA, Crawford EA, Andel R, McBride NM, et al. A randomized controlled trial in community mental health centers of computer-assisted cognitive behavioral therapy versus treatment as usual for children with anxiety. Depress Anxiety. 2015;32(11):843–52. 26366886 10.1002/da.22399

[34] Vigerland S, Ljótsson B, Thulin U, Öst L-G, Andersson G, Serlachius E. Internet-delivered cognitive behavioural therapy for children with anxiety disorders: a randomised controlled trial. Behav Res Ther. 2016;76:47–56. 26649465 10.1016/j.brat.2015.11.006

[35] Waite P, Marshall T, Creswell C. A randomized controlled trial of internet-delivered cognitive behaviour therapy for adolescent anxiety disorders in a routine clinical care setting with and without parent sessions. Child Adolesc Mental Health. 2019;24(3):242–50. 10.1111/camh.12311 32677216

[36] Wuthrich VMPD, Rapee RMPD, Cunningham MJPD, Lyneham HJPD, Hudson JLPD, Schniering CAPD. A randomized controlled trial of the cool teens CD-ROM computerized program for adolescent anxiety. J Am Acad Child Adolesc Psychiatry. 2012;51(3):261–70. 22365462 10.1016/j.jaac.2011.12.002

[37] López-Soler C, Vicente-Escudero JL, López-López JA, Alcántara M, Martínez A, Castro M, et al. Effectiveness of internet-delivered psychological treatments for children and adolescents with anxiety and/or depressive disorders: systematic review and network meta-analysis. Int J Clin Health Psychol. 2024;24(3):100487. 39114408 10.1016/j.ijchp.2024.100487 PMC11304886

[38] Harris PA, Taylor R, Minor BL, Elliott V, Fernandez M, O’Neal L, et al. The REDCap consortium: building an international community of software platform partners. J Biomed Inform. 2019;95:103208. 31078660 10.1016/j.jbi.2019.103208 PMC7254481

[39] Harris PA, Taylor R, Thielke R, Payne J, Gonzalez N, Conde JG. Research electronic data capture (REDCap)—a metadata-driven methodology and workflow process for providing translational research informatics support. J Biomed Inform. 2009;42(2):377–81. 18929686 10.1016/j.jbi.2008.08.010 PMC2700030

[40] Andersson G, Paxling B, Wiwe M, Vernmark K, Felix CB, Lundborg L, et al. Therapeutic alliance in guided internet-delivered cognitive behavioural treatment of depression, generalized anxiety disorder and social anxiety disorder. Behav Res Ther. 2012;50(9):544–50. 22728647 10.1016/j.brat.2012.05.003

[41] Cuijpers P, Donker T, van Straten A, Li J, Andersson G. Is guided self-help as effective as face-to-face psychotherapy for depression and anxiety disorders? A systematic review and meta-analysis of comparative outcome studies. Psychol Med. 2010;40(12):1943–57. 20406528 10.1017/S0033291710000772

[42] Bennett SD, Cuijpers P, Ebert DD, McKenzie Smith M, Coughtrey AE, Heyman I, et al. Practitioner review: unguided and guided self-help interventions for common mental health disorders in children and adolescents: a systematic review and meta-analysis. J Child Psychol Psychiatry. 2019;60(8):828–47. 30775782 10.1111/jcpp.13010

[43] Smart K, Smith L, Harvey K, Waite P. The acceptability of a therapist-assisted internet-delivered cognitive behaviour therapy program for the treatment of anxiety disorders in adolescents: a qualitative study. Eur Child Adolesc Psychiatry. 2023;32(4):661–73. 34746976 10.1007/s00787-021-01903-6 PMC8572655

[44] Kendall PC. Treating anxiety disorders in children: results of a randomized clinical trial. J Consult Clin Psychol. 1994;62(1):100. 8034812 10.1037//0022-006x.62.1.100

[45] Rapee RM, Lyneham HJ, Schniering CA, Wuthrich V, Abbott MA, Hudson JL, et al. Cool Kids: Child and adolescent anxiety program. Sydney: Centre for Emotional Health, Macquarie University; 2006.

[46] Spence SH. A measure of anxiety symptoms among children. Behav Res Ther. 1998;36(5):545–66. 9648330 10.1016/s0005-7967(98)00034-5

[47] Arendt K, Hougaard E, Thastum M. Psychometric properties of the child and parent versions of Spence Children’s Anxiety Scale in a Danish community and clinical sample. J Anxiety Disord. 2014;28(8):947–56. 25445085 10.1016/j.janxdis.2014.09.021

[48] McLellan LF, Kangas M, Rapee RM, Iverach L, Wuthrich VM, Hudson JL, et al. The Youth Online Diagnostic Assessment (YODA): validity of a new tool to assess anxiety disorders in youth. Child Psychiatry Hum Dev. 2021;52(2):270–80. 32440754 10.1007/s10578-020-01007-3

[49] Silverman WK, Albano AM. Anxiety disorders interview schedule for DSM-IV: child version. New York: Oxford University Press; 1996.

[50] Lyneham HJ, Sburlati ES, Abbott MJ, Rapee RM, Hudson JL, Tolin DF, et al. Psychometric properties of the Child Anxiety Life Interference Scale (CALIS). J Anxiety Disord. 2013;27(7):711–9. 24135256 10.1016/j.janxdis.2013.09.008

[51] Daviss BW, Birmaher B, Melhem NA, Axelson DA, Michaels SM, Brent DA. Criterion validity of the mood and feelings questionnaire for depressive episodes in clinic and non-clinic subjects. J Child Psychol Psychiatry. 2006;47(9):927–34. 16930387 10.1111/j.1469-7610.2006.01646.x

[52] Eg J, Bilenberg N, Costello EJ, Wesselhoeft R. Self- and parent-reported depressive symptoms rated by the mood and feelings questionnaire. Psychiatry Res. 2018;268:419–25. 30130708 10.1016/j.psychres.2018.07.016

[53] Derrett S, Herdman M, Ngwira LG, Moore EY, Jelsma J. A new approach to assessing children’s interpretation of severity qualifiers in a multi-attribute utility instrument-the EQ-5D-Y-5L: development and testing. Patient Patient Cent Outcomes Res. 2021;14(5):591–600. 10.1007/s40271-021-00496-1 PMC8357732 33650034

[54] Herrero R, Vara MD, Miragall M, Botella C, García-Palacios A, Riper H, et al. Working alliance inventory for online interventions-short form (Wai-tech-sf): the role of the therapeutic alliance between patient and online program in therapeutic outcomes. Int J Environ Res Public Health. 2020;17(17):6169. 32854381 10.3390/ijerph17176169 PMC7503297

[55] Hatcher RL, Gillaspy JA. Development and validation of a revised short version of the working alliance inventory. Psychother Res. 2006;16(1):12–25.

[56] Larsen DL, Attkisson CC, Hargreaves WA, Nguyen TD. Assessment of client/patient satisfaction: development of a general scale. Eval Program Plann. 1979;2(3):197–207. 10245370 10.1016/0149-7189(79)90094-6

[57] Attkisson CC, Greenfield TK. Client satisfaction questionnaire-8 and service satisfaction scale-30. 1994.

[58] Brooke J. SUS: a “quick and dirty” usability scale. In: Thomas JBW, B, editor. Usability evaluation in industry. London: Taylor & Francis; 1996. p. 189–94.

[59] Hvidt JCS, Christensen LF, Sibbersen C, Helweg-Jørgensen S, Hansen JP, Lichtenstein MB. Translation and validation of the system usability scale in a Danish mental health setting using digital technologies in treatment interventions. Int J Hum Comput Interact. 2020;36(8):709–16.

[60] Rozental A, Kottorp A, Forsström D, Månsson K, Boettcher J, Andersson G, et al. The Negative Effects Questionnaire: psychometric properties of an instrument for assessing negative effects in psychological treatments. Behav Cogn Psychother. 2019;47(5):559–72. 30871650 10.1017/S1352465819000018

[61] Spence SH, Sawyer MG, Sheffield J, Patton G, Bond L, Graetz B, et al. Does the absence of a supportive family environment influence the outcome of a universal intervention for the prevention of depression? Int J Environ Res Public Health. 2014;11(5):5113–32. 24828082 10.3390/ijerph110505113 PMC4053893

[62] Angold A, Costello EJ, Messer SC, Pickles A. Development of a short questionnaire for use in epidemiological studies of depression in children and adolescents. Int J Methods Psychiatr Res. 1995.

[63] Eyre O, Jones RB, Agha SS, Wootton RE, Thapar AK, Stergiakouli E, et al. Validation of the short mood and feelings questionnaire in young adulthood. J Affect Disord. 2021;294:883–8. 34375216 10.1016/j.jad.2021.07.090 PMC8411664

[64] Leon AC, Heo M. Sample sizes required to detect interactions between two binary fixed-effects in a mixed-effects linear regression model. Comput Stat Data Anal. 2009;53(3):603–8. 20084090 10.1016/j.csda.2008.06.010 PMC2678722

[65] Hsieh F. A simple method of sample size calculation for unequal-sample-size designs that use the logrank or t-test. Stat Med. 1987;6(5):577–81. 3659667 10.1002/sim.4780060506

[66] StataCorp. Stata statistical software: release 18. College Station: StataCorp LLC; 2023.

[67] Food Drug Administration International conference on harmonization. Good clinical practice: consolidated guidelines. Fed Reg. 1997;62:25692–709.

